# Development and Delivery of an Integrated Digital Health Care Approach for Children With Juvenile Idiopathic Arthritis: Usability Study

**DOI:** 10.2196/56816

**Published:** 2024-09-17

**Authors:** Sonia Butler, Dean Sculley, Derek Santos, Xavier Gironès, Davinder Singh-Grewal, Andrea Coda

**Affiliations:** 1 School of Bioscience and Pharmacy, College of Health, Medicine and Wellbeing, University of Newcastle, Ourimbah, Australia; 2 School of Health Sciences, Queen Margaret University, Edinburgh, United Kingdom; 3 Department of Research and Universities, Generalitat de Catalunya, Government of Catalonia, Barcelona, Spain; 4 Department of Rheumatology, Sydney Children's Hospitals Network, Randwick and Westmead, Sydney, Australia; 5 Discipline of Child and Adolescent Health, University of Sydney, Sydney, Australia; 6 School of Women’s and Children’s Health, University of New South Wales, Sydney, Australia; 7 Department of Rheumatology, John Hunter Children’s Hospital, Newcastle, Australia; 8 School of Health Sciences, College of Health, Medicine and Wellbeing, University of Newcastle, Ourimbah, Australia; 9 Equity in Health and Wellbeing Research Program, Hunter Medical Research Institute, Newcastle, Australia

**Keywords:** phone app, smart watch, juvenile idiopathic arthritis, pain, medication adherence, physical activity, integrated care, medication, development, usability study, chronic inflammatory disorder, children, child, usability, survey, thematic analysis, gamification, modules, web-based platform, support

## Abstract

**Background:**

Juvenile idiopathic arthritis (JIA) is a chronic inflammatory disorder with no cure. Most children are prescribed several medications aimed at controlling disease activity, managing symptoms, and reducing pain. Physical activity is also encouraged to retain musculoskeletal function. The primary determinants of treatment success are maintaining long-term adherence, ongoing monitoring by a pediatric rheumatologist, and involvement of an interdisciplinary team. To support these goals, a new digital intervention was developed, InteractiveClinics, which aimed to prompt children to take their medications, report pain levels, and increase their physical activity.

**Objective:**

This study aims to evaluate the usability of InteractiveClinics among children with JIA.

**Methods:**

As part of this pediatric cross-sectional study, 12 children were asked to wear a smartwatch for 2 weeks, which was synchronized to the InteractiveClinics phone app and web-based platform. Personalized notifications were sent daily to the watch and phone, to prompt and record medication adherence and pain level assessment. Physical activity was automatically recorded by the watch. At the end of the study, all children and parents completed a postintervention survey. Written comments were also encouraged to gain further feedback. Descriptive statistics were used to summarize the survey results, and all qualitative data underwent thematic analysis.

**Results:**

Twelve children aged 10 to 18 years (mean 14.2, SD 3.1 years; female: n=8, 66.7%) and 1 parent for each child (n=12; female: n=8, 66.7%) were enrolled in the study. Based on the highest and lowest agreement areas of the survey, most children and parents liked the smartwatch and web-based platform; they found it easy to learn and simple to use. They were also satisfied with the pain and physical activity module. However, usability and acceptability barriers that hindered uptake were identified in the phone app and medication module. Children required a unique in-app experience, and their suggestive improvements included more personalization within the app; simplification by removing all links not relevant to antirheumatic medications; flexibility in response times; improved conferment through gamification; additional comment fields for the input of more data, such as medication side effects or pain-related symptoms; more detailed graphical illustrations of the physical activity module, including a breakdown of metrics; and importantly, interconnections between modules, because medication adherence, pain levels, and physical activity can each influence the other. They were, overall, improving usefulness for children and parents.

**Conclusions:**

The usability of InteractiveClinics was positive. Children and parents liked the watch and web-based platform and were satisfied with the pain and physical activity module. However, children wanted a unique in-app experience through more personalization, simplification, flexibility, conferment, comment fields, graphical illustrations, a breakdown of metrics, and interconnections. Certainly, inclusions are needed to promote user adoption and advancement of new validated digital health interventions in pediatric rheumatology, to support the delivery of integrated care.

## Introduction

### Overview

Globally, more than 3 million children are currently living with the autoimmune disorder juvenile idiopathic arthritis (JIA) [1,2]. JIA is the broad term used to describe a heterogeneous group of 7 inflammatory disorders, all of which have an unknown origin that begins in children younger than 18 years [3,4]. The commonality between these disorders is joint inflammation and pain [5,6]. Prolonged exposure to this inflammation can cause serious widespread complications for a child, such as impaired growth [7,8], delayed pubertal development [8,9], premature cardiovascular disease [10], and organ damage [11]. Ongoing exposure to pain can induce permanent changes in the nervous system, increasing pain sensitivity and the continuance of persistent pain in adulthood [6,12]. Regrettably, there is no cure. Instead, a “treat to target“ approach is used to induce clinical remission or lower disease activity [13]. The aim is to normalize and preserve joint function, prevent growth retardation, maintain physical function, and avoid permanent disability [7,14], ultimately improving the child’s quality of life [15].

### Background

#### Medications

For most children, the first line of treatment includes the aggressive use of medications, for a prolonged period of time, because the therapeutic response to gain disease control and symptom relief is slow [16,17]. Typically, these medications include disease-modifying antirheumatic medications (such as methotrexate, sulphasalazine, and leflunomide) in combination with corticosteroids to target inflammation. In addition, ibuprofen and naproxen can ease the pain and further calm the swelling [14,16,18]. However, adhering to a strict medication schedule can be difficult for many children due to the need to take multiple medications of varying doses, by different routes of administration (oral, intravenous, and intra-articular) [14,16,19] and on different days at different times, to limit the medications’ side effects on well-being [20]. An example is administering disease-modifying antirheumatic medications on a Friday night, to ensure the associated nausea and brain fog do not interfere with school performance. Therefore, reliable monitoring of a child’s medication adherence by a pediatric rheumatologist is crucial to ensure they are gaining the full therapeutic response and alleviating any side effects they may experience [21].

#### Pain Level Monitoring

Pain is often one of the first JIA-related symptoms experienced by a child. Pain begins at an early stage of the disease trajectory when a child’s joints begin to swell and become restricted. It is at this early stage that a child’s pain perception can be permanently altered [6,12] because pain activates changes in the central and peripheral neural pathways, decreasing a child’s pain threshold [22] and increasing sensitization [12]. This means pain can persist despite good disease control [6,23] and become widespread throughout the body, such as in both the affected and unaffected knee [24]. Understandably, it is essential that pain fluctuations for JIA are meticulously monitored, and appropriate treatments quickly initiated to achieve pain-free remission [12,15].

#### Physical Activity

Exercise is prescribed for children with JIA because of the abundant health benefits [25]. Exercise helps to retain musculoskeletal function and muscle strength [26], cardio-respiratory health, a healthy weight, and good mental health [27]. Yet, children are often not motivated to exercise because of their symptoms, such as chronic synovial joint inflammation, erythema, pain, stiffness, limited range of movement, and fatigue [28]. However, this motivation does not change when there is no pain and good disease control [29]. Perhaps this is because many children and parents are under the belief that exercise will exacerbate symptoms [25], when the reverse is true, exercise can promote alleviation. In fact, a recent systematic review reported no adverse events related to exercise [25].

Importantly, there are also many non–JIA-related reasons why children do not exercise. They simply do not enjoy it, have too little time, or need parental support to attend exercise programs [30] or pay for the ongoing costs associated with attending [31]. Undeniably, in order to improve the current low adherence rates for children with JIA (40%‐47%) [32,33], a new, cost-effective way is needed to promote and maintain a regular exercise regime.

#### Interdisciplinary Team

To achieve the best possible outcomes for children with JIA, children need access to a diverse interdisciplinary team that works in conjunction with the pediatric rheumatologist, child, and parents [34-36]. Services from allied health can include physiotherapy, occupational therapy, and podiatry to support physical function and pain [36-38], while nursing can support medication adherence through education and demonstration of injection techniques [36]. Importantly, this support also needs to be maintained for an extended period of time [39], and ongoing monitoring of the child is needed to ensure treatment success [37].

#### Digital Health

Recent advances in smart technology have the potential to support chronic disease management [40,41] by improving treatment adherence, recording symptoms, and monitoring health behaviors [42]. For JIA, digital health innovations could be adopted to prompt medication adherence, record pain levels, and improve physical activity. These are 3 key areas that can lead to poor outcomes. Research to date on eHealth and mobile health (mHealth) interventions for JIA has predominantly focused on electronic pain diaries and web-based programs [43]. These electronic pain diaries have used personal digital assistants [44], Apple iPods, and computers [45], while web-based programs have focused on providing education and skills to promote chronic disease management, cognitive behavior [46], physical activity [47], peer support [48], and improved quality of life [49]. Overall, feasibility and usability studies have revealed that these forms of interventions are readily accepted by children [45,46]. They find this technology easy to use and navigate [50] and need little training because they are already using the internet and mobile technology in their day-to-day life [51,52]. In fact, in Australia, 81% of children over 14 years and 55% between 10 and 13 years own a mobile phone [53,54]. Internationally, 18%‐43% of children aged between 13 and 17 years and 7%‐45% of children aged 6‐12 years own a smartwatch [55], with a predicted rate that will substantially increase over the next few years [56].

#### InteractiveClinics

InteractiveClinics is an innovative digital health web-based platform aimed at supporting digital health research. It was developed by academics from the University of Newcastle, Australia, and the University of Manresa (Catalonia) Spain, with IT support from BitGenoma Ltd Digital Solutions. For JIA, InteractiveClinics was used to prompt and monitor the 3 modifiable risk factors associated with poor JIA-related outcomes—medication adherence, pain levels, and physical activity—by using a commercially available smartwatch, a customized phone app, and a password-protected Australian web server. To further address the ethical and privacy issues related to data safety, all data collected by the app and web-based platform was managed in accordance with the University of Newcastle’s Information Security Data Classification and Handling Manual, and the Privacy Management Plan. The cost of development for the platform, app, and important server protection was approximately Aus $75,000 (US $48,000).

### Objectives

The aim of this study is to evaluate the usability and acceptability of InteractiveClinics among children (aged 10‐18 years) by determining if the intervention is (1) easy to use, (2) acceptable, and (3) useful [57,58].

## Methods

### Study Overview

This study was part of a cross-sectional study, following the World Health Organization’s 6-stage step-up approach to develop a digital health intervention. These steps start from monitoring the intervention’s functionality and fidelity through to evaluating feasibility, usability, efficacy, and effectiveness [58], allowing improvements to be put into place after each stage of testing and improving the quality of the intervention [59].

This study focuses on evaluating InteractiveClinics’ usability to support the development of a user-centered design, because the success of an intervention is dependent on whether intended end users engage with the intervention [58]. For 2 weeks, children and parents gained hands-on experience using InteractiveClinics and then completed a postintervention survey. To ensure detailed analysis, survey questions were based on a quantitative descriptive and qualitative design, to invite participants to answer questions in their own words [60].

### Recruitment of Participants

As part of this pediatric cross-sectional study, children were recruited through 2 pediatric rheumatology outpatient clinics within 2 tertiary children’s hospitals in Australia. The eligibility criteria included an age range of 10 to 18 years, a diagnosis of JIA, and good comprehension of the English language. The exclusion criteria included a cognitive impairment, physical disability, or visual impairment that would affect the child’s ability to understand or use smart technology.

### Ethical Considerations

Ethics approval for this study was granted by the Hunter New England Research Ethics Committee (approval no: 2019/ETH01035). To ensure informed consent, all potential participants were provided with an information sheet, explaining the study’s purpose, expectations, and how all data collected will be deidentified. Additionally, all study participants were informed that they could withdraw from the study at any time, without discrimination, by simply not completing the survey. In total, 12 children and 12 parents agreed to participate in the study, provided signed consent, and completed the anonymous survey between September and November 2022 (Table 1).

**Table 1.** Participants’ demographics.

Demographics		Values
**JIA subtype** ^**a**^ **, n (%)**		
	Polyarthritis (rheumatoid factor [Rh] negative)	5 (41.7)
	Oligoarthritis	4 (33.3)
	Enthesitis related	1 (8.3)
	Polyarthritis (Rh positive)	1 (8.3)
	Psoriatic	1 (8.3)
**Medications, n (%)**		
	DMARDs^b^	5 (41.7)^c^
	bDMARDs^d^	2 (16.7)
	NSAIDs^e^	5 (41.7)
	Corticosteroids	3 (25)
	Pain relievers	7 (58)
	Folic acid	1 (8.3)
Disease duration (years), mean (range)		4.9 (5 months to 10 years)

a Juvenile idiopathic arthritis (JIA) subtypes based on the International League of Associations for Rheumatology criteria [3].

b DMARD: disease-modifying antirheumatic drug.

c One participant was prescribed both DMARDs and bDMARDs.

d bDMARD: biological disease-modifying antirheumatic drug.

e NSAID: nonsteroidal anti-inflammatory drug.

### Intervention

**Figure 1.**
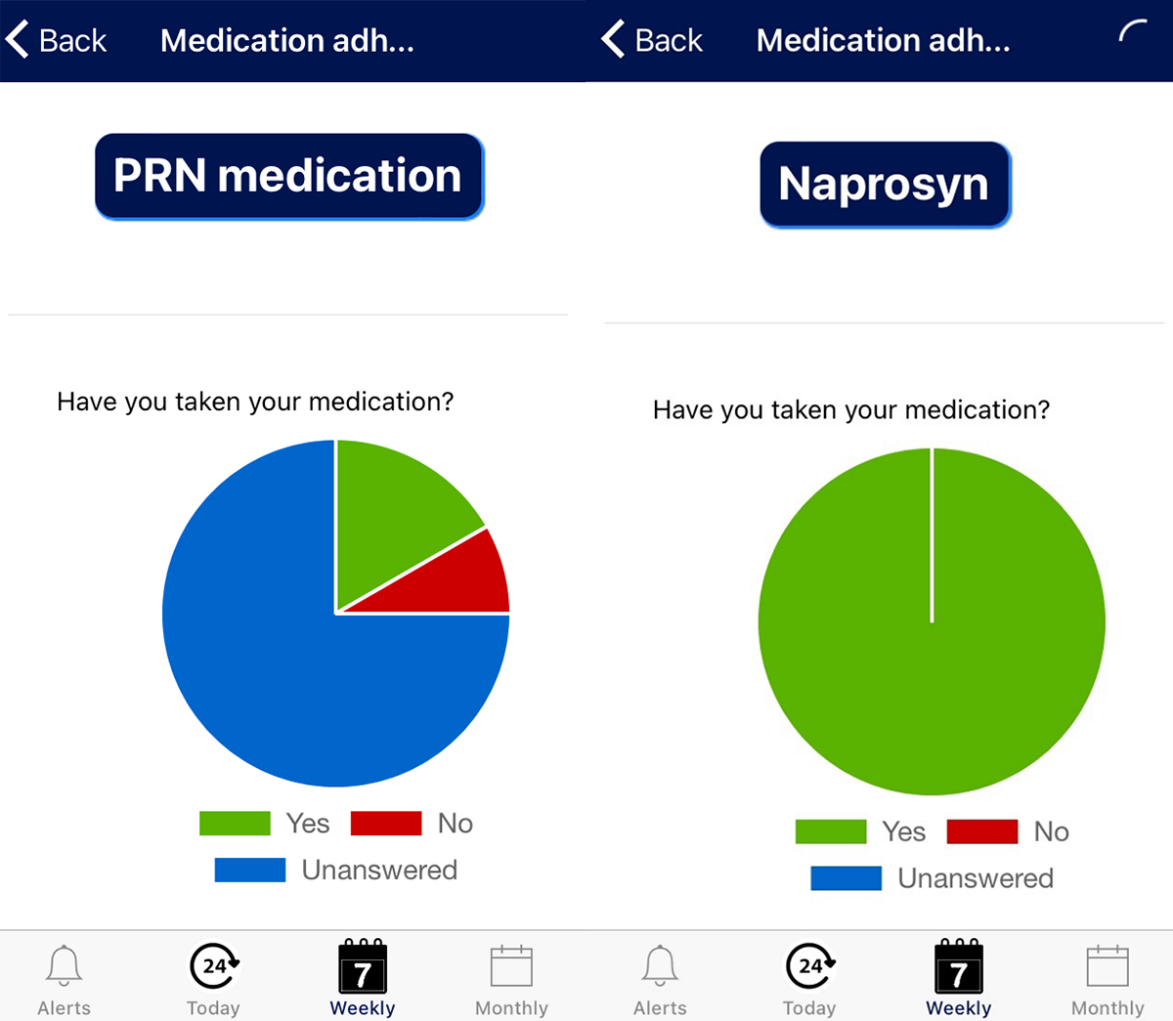
Weekly example of medication adherence responses.

**Figure 2.**
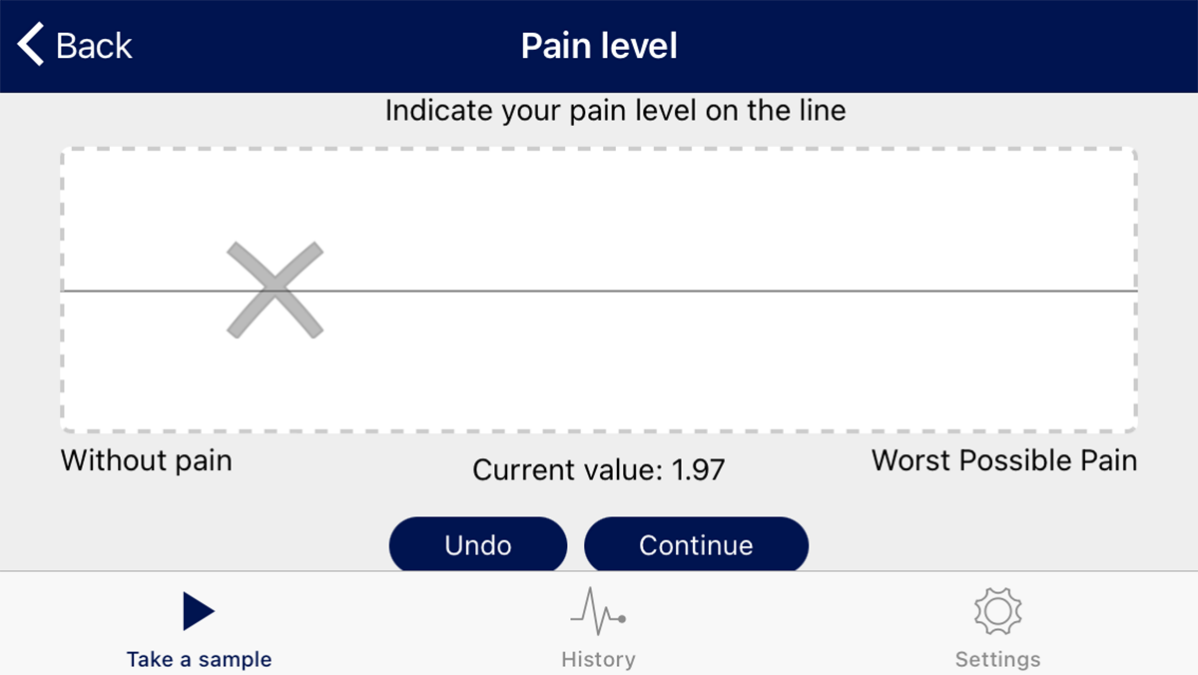
Recording pain level on the electronic visual analogue scale module.

InteractiveClinics aims to motivate children to take their medication, record their pain, and increase their participation in physical activity. Personalized notifications were sent daily to the smartwatch and phone, to prompt and record medication adherence (Figure 1) and complete a pain level assessment within the app (Figure 2). Physical activity was automatically recorded by simply wearing the watch.

InteractiveClinics presents these 3 key areas of monitoring as 3 modules—medication adherence, pain level, and physical activity level—which can be monitored daily, weekly, or monthly by the child within the app (Figures1  3) or on a secure, password-locked, web-based platform by the child, parents/caregiver, pediatric rheumatologist, and health care team (Figure 4).

Pain levels were recorded on the validated electronic visual analog scale (eVas) module [61-63]. eVas uses a simple horizontal line with defined pain limits. The left end point indicates “without pain,” and the right end point indicates “worst possible pain” (Figure 2). This reporting scale has been found to be highly reliable and consistent with the original paper-based visual analog scale [61-63].

**Figure 3.**
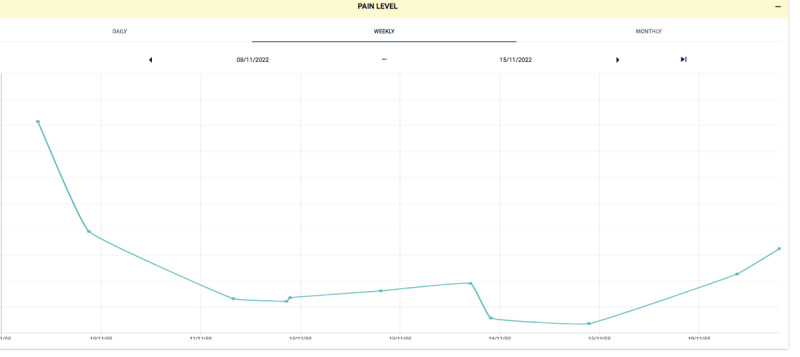
Weekly example of real-time pain levels.

**Figure 4.**
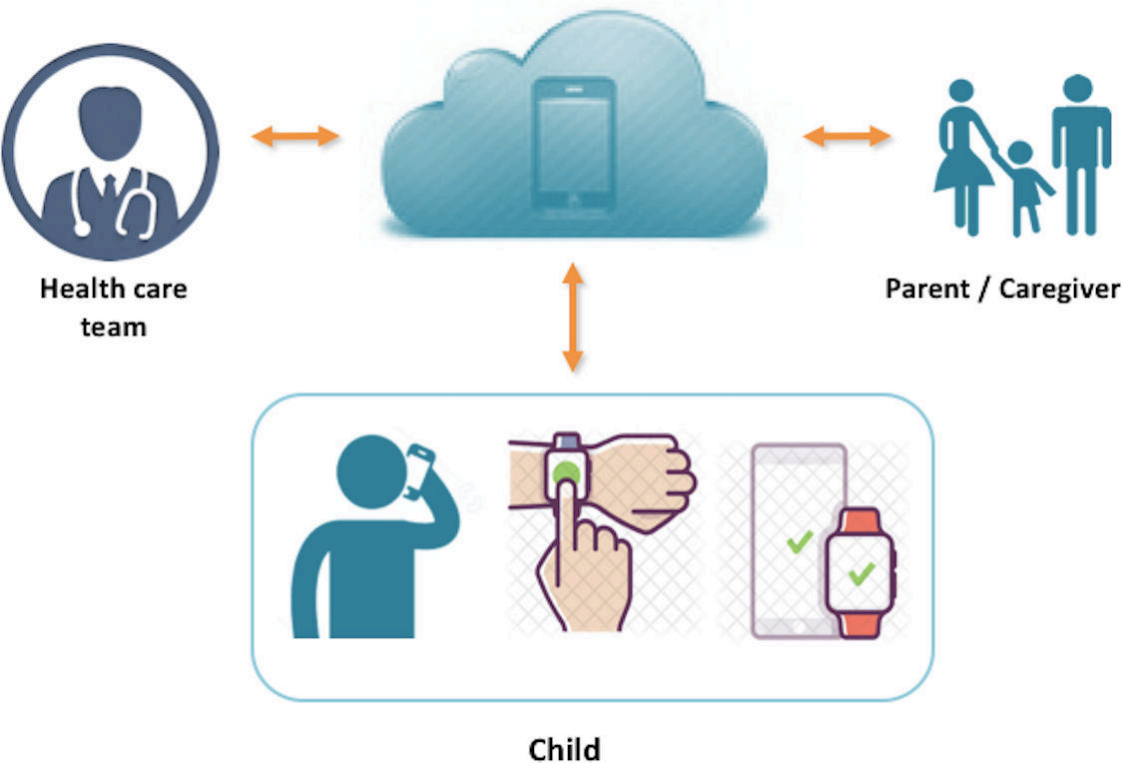
Data communication supporting an integrative model of care for juvenile idiopathic arthritis.

### Materials

The materials supplied to support participation in the intervention were as follows: a smartwatch (Apple Watch series 3, with a water-resistance rating of 50 meters, to support low-intensity activities such as swimming [64]), a smartphone (Apple iPhone, SE, 2016, loaded with Aus $30 [US $20.01] worth of pre-paid credit), the InteractiveClinics app (preset on the iPhone), and a personal password (to access the data collected on the secure web-based platform).

The persuasive influences used to promote the adoption of the intervention were as follows [43]: training (for 15 minutes on how to use the smartwatch, InteractiveClinics app, and web-based platform), an instruction manual, ongoing technical support, ongoing human communication (researcher contact details [SB] were placed in the phone), goals set by the user (for the physical activity module), personal reminders (daily personal notification sent to the watch and phone, at a time preselected by child, to prompt and record medication administration and pain level assessment), and a reactive feedback loop (for the physical activity module).

A more detailed description of InteractiveClinics has been provided in an earlier feasibility study [59].

### Data Collection

InteractiveClinics’ usability and acceptability were measured through a postintervention survey. Two surveys were developed to collect feedback from children and their parents after they used the digital intervention for 2 weeks. Survey questions were adapted from the System Usability Scale [65,66], to ensure the survey questions encompassed the unique multimodal approach being used by InteractiveClinics. Further, to enable younger children to complete the survey without help from their parents, age-appropriate language was used.

For the parents, survey responses were based on the 1-to-5 Likert scale (1=strongly disagree, 2=disagree, 3=unsure, 4=agree, and 5=strongly agree). For children, survey responses were based on the visual face scale [66].

Written comments were also encouraged by children and parents throughout the survey, in a free-text format, to identify any unmet inclusions; to illustrate important points; and, importantly, to facilitate a user-friendly design.

### Data Analysis

#### Quantitative Data Analysis

Descriptive statistics were used to summarize participants’ demographic characteristics. For the survey results, a percentage agreement was used to place the data into 2 independent judgments, to allow the examination of either agreement or disagreement [67]. Commonly agreed included the responses “strongly agree” and “agree,” and commonly disagreed included the responses *“*unsure,” “disagree,” and “strongly disagree.” This form of data analysis focuses on reporting the proportion of answers that agree, by calculating them as a percentage [68]. These percentages can then be easily compared because there is a common denominator.

#### Qualitative Data Analysis

For all the qualitative data, this study used thematic analysis. This is an inductive approach used to examine themes and patterns within the data [69]. Coding began by clustering together small descriptive segments to expose both the strengths and weaknesses of InteractiveClinics. Then collectively, mutual patterns emerged forming latent themes, allowing the child’s and their parent’s experience to be heard.

To ensure rigor at all stages of interpretation, codes were continually checked to ensure they retained their original meaning, and trustworthiness and clinical relevance were enhanced by all members of our research team. Final results were internally reviewed, and data saturation was reached, drawing no more conclusions.

## Results

### Child Survey Feedback

The response rate to the survey among children was 100% (12/12). Most children (9/12, 75%) also completed the survey without any help from their parents. The results of the quantitative section of the survey are presented in Table 2.

**Table 2.** Quantitative survey results: child version (n=12).

Child survey questions		Smartwatch, n (%) agree	App, n (%) agree	Web-based platform, n (%) agree
**Usability**				
	Easy to learn	12 (100)	6 (50)	7 (58.3)
	Simple to use	12 (100)	8 (66.7)	9 (75)
	Felt comfortable using the…	11 (92)	8 (66.7)	8 (66.7)
	Quick to use	9 (75)	9 (75)	10 (83.3)
	Information clear and well organized	8 (67)	6 (50)	7 (58.3)
**Acceptability**				
	I liked the…	9 (75)	3 (25)	8 (66.7)
	Was fun	9 (75)	0 (0)	2 (16.7)
	Was useful	10 (83)	5 (41.7)	6 (50)
	Satisfied with…	9 (75)	6 (50)	6 (50)
	Would recommend to other young people with arthritis	10 (83)	5 (41.7)	5 (41.7)

#### Smartwatch

Based on the highest and lowest agreement areas of the survey, the results support using a smartwatch as part of the intervention. The watch was easy to learn (12/12, 100%) and simple to use (12/12, 100%), and the information was useful (10/12, 83%). However, only 8 children reported the information was clear and organized (8/12, 67%). Written feedback also identified children were “expecting the App to open in the watch.” Instead they “could not answer anything on the watch.” Children wanted an expandable notification with a reply action, to allow their medication administration and pain levels to be directly recorded from the watch.

#### Phone App

In comparison to the smartwatch, problems were identified within the phone app.

Although the perceived usability of the app was reported as simple (8/12, 66.7%) and quick (9/12, 75%), only half of the participants found learning to use the app easy (6/12, 50%). Furthermore, only a small number of children liked the app (3/12, 25%), and none described the app as fun (0/12, 0%), impairing acceptability. Written feedback revealed participants were seeking a unique in-app experience through demographic *“*personalisation*.”*

#### Web-Based Platform

The usability of the web-based platform was reported as simple (9/12, 75%) and quick (10/12, 83.3%). However, only 7 participants reported it was easy to learn (7/12, 58.3%). Similar to the app, only half of the participants reported the platform as useful (6/12, 50%) or were satisfied (6/12, 50%). Further, only 2 participants reported the platform to be fun (2/10, 16.7%). Written feedback resulted in the following theme: interconnections. Participants wanted to see a comparison between the 3 modules, medication adherence, pain levels, and physical activity, to explore if any relationships exist. A participant explained the following: “has the potential to be helpful if I could access the physical activity [and medication adherence] results in line with my pain” (child 7).

### Parent Survey Feedback

The response rate to the survey among parents was 100% (12/12). The results of the quantitative section of the survey are presented in Table 3.

**Table 3.** Quantitative survey results: parent version (n=12).

Parents survey questions	Smartwatch, n (%) agree	App, n (%) agree	Web-based platform, n (%) agree
Easy to learn	11 (91.7)	8 (66.7)	7 (58.3)
Simple to use	11 (91.7)	9 (75)	8 (66.7)
Comfortable in supporting my child	11 (91.7)	11 (91.7)	10 (83.3)
Supporting my child was quick	10 (83.3)	8 (66.7)	8 (66.7)
I did not need to prompt my child	7 (58.3)	5 (41.7)	3 (25)
My child was independent	9 (75)	8 (66.7)	5 (41.7)
Information is clear and well organized	10 (83.3)	8 (66.7)	7 (58.3)
The system is error-free	10 (83.3)	3 (25)	10 (83.3)

#### Smartwatch

Most parents’ responses supported the usability and acceptability of the smartwatch. Parents reported the watch was easy to learn (11/12, 91.7%) and simple to use (11/12, 91.7%). However, only 9 parents reported children could independently use the watch (9/12, 75%), and 7 parents did not need any prompting (7/12, 58.3%). No written feedback for improvement was recorded.

#### Phone App

Most parents felt comfortable supporting their child (11/12, 91.7%) and agreed using the app was simple (9/12, 75%). However, only a small number of parents did not need to prompt their child to use the app (5/12, 41.7%), or found the system error-free (3/12, 25%).

I don’t think the app is working properly. [parent 5]

To improve the app, written feedback suggested simplification, to ensure the app directly aligned with their child’s needs. A parent explained the following: “simplified interface by removal of unnecessary buttons” (parent 6).

#### Web-Based Platform

Parents reported that they were comfortable in supporting their child when using the platform (10/12, 83.3%), and found the system to be error-free (10/12, 83.3%). However, less than half reported that their child could independently use the platform (5/12, 41.7%), and only 3 parents did not need to prompt their child (3/12, 25%). Suggested improvements included “improving the activity rings [for the physical activity module]” (parent 3).

### Modules Within the App

#### Medication Adherence Module

##### Child Usability

Half of the participants agreed medication reminders were helpful (6/12, 50%), sent at the right time (6/12, 50%), and not bothersome (7/12, 58.3%). There were also no reported adverse events (12/12, 100%).

##### Child Acceptability

Less than half of the participants were satisfied with the medication module (5/12, 41.7%). Even less agreed that the response list was clear (2/12, 16.7%). Only 3 children would continue to use the medication module (3/12, 25%). Suggestions for improvement included flexibility. Rather than asking the research team to update their medications, children wanted to “self-change medication times [within the app]” (child 2) and “add or remove medications [within the app]” (child 12).

##### Parent Satisfaction

Parents were also not satisfied with the medication module (5/12, 41.7%), resulting in the following theme: comment fields. They wanted to see a broader range of data collected that aligned with their child’s needs. For example:

*add comment fields* to record *medication symptoms such as nausea from Methotrexate, headaches from Humira, exhaustion and brain fog.* [parent 9]

### Pain Level Module

#### Overview

Children agreed recording pain was helpful (8/12, 66.7%), how to record their pain was clear (10/12, 83.3%), and responding to the pain reminders was not bothersome (7/12, 58.3%). There were also no adverse events reported (12/12, 100%). However, less than half were satisfied (4/12, 33.3%) and would continue using the pain module (6/12, 50%). Children were underwhelmed by the eVas response to their pain. When they entered their pain level on the numerical line and pressed confirm, only the numerical value between 0 and 10 emerged, describing their pain.

When you put your pain in nothing happens. [child 1]

This resulted in the following theme: conferment. Children suggested that the pain score should include gamification or the use of “visual aids, which may be of benefit.”

Similar to the feedback from the medication module, children also wanted flexibility. Although pain scores could be added at any time in the app, children wanted to be able to self-adjust their pain-reporting notification time within the app.

*I wanted to change my pain time.* [child 5]

#### Parent Satisfaction

Most parents were satisfied with the pain module (8/12, 66.7%). However, parents expressed, again, wanting to *“*record*”* additional information. Further supporting the comment fields theme, this would allow them to document “joint/s the pain is in” (parent 3) and “potential contributing factors for pain such as weather, over-exertion” (parent 9).

### Physical Activity Module

#### Child Usability

Children were overall satisfied with the physical activity module (9/12, 75%). Most followed (8/12, 66.7%) and understood their physical activity levels (8/12, 66.7%) and agreed that the module increased their physical activity (7/12, 58.3%).

#### Child Acceptability

Half the children agreed that recording their physical activity was helpful (6/12, 50%) and would like to continue using the physical activity intervention (6/12, 50%). No adverse events were reported (12/12, 100%).

Written feedback suggesting how to improve this module included a graphical illustration. Children reported they needed more graphical representation of their daily and weekly physical activity levels.

*Have the physical activity show more details* [child 1]

#### Parent Satisfaction

More than half of the parents were satisfied with the physical activity module (7/12, 58.3%), and their feedback on improvements aligned with children, suggesting more details, such as “a break-down of metrics” (parent 7).

### Overall Satisfaction With InteractiveClinics

Overall, most children found all the information on InteractiveClinics easy to understand (9/12, 75%). However, only 5 children reported that the information collected met their needs (5/12, 41.7%), and 4 children reported that it would support their doctor with their care (4/12, 33.3%).

In contrast, most parents felt InteractiveClinics was appropriate to address their child’s needs (10/12, 83.3%). They found the information useful (8/12, 66.7%) in supporting their understanding of the effects JIA had on their child (8/12, 66.7%) and would use InteractiveClinics again (6/12, 50%). Importantly, 7 parents reported that InteractiveClinics would support their child in their next pediatric rheumatology consultation (7/10, 58.3%).

We found the App very useful. My child required no prompting to record her pain levels and it was good for me to understand her level of pain.

[parent 6]

I think it will be very useful in the future.

[parent 4]

**Figure 5.**
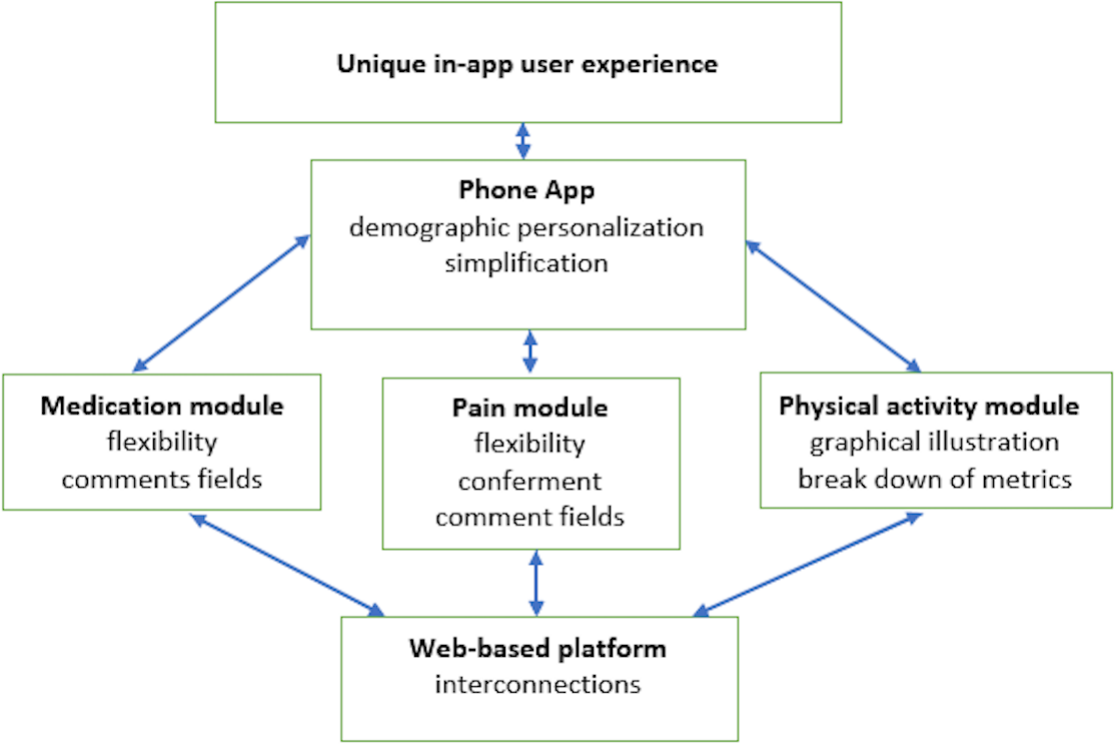
Coding tree representing the analyzed content.

Summing up the feedback on all key areas in InteractiveClinics*,* participants wanted a unique in-app experience (Figure 5).

## Discussion

### Principal Findings

This study sought to evaluate the usability and acceptability of InteractiveClinics for children (aged 10 to 18 years) living with JIA. A unique feature of InteractiveClinics is the multimodal approach it uses to support the 3 key management areas in JIA treatment: medication adherence, pain, and the retention of physical activity. InteractiveClinics was supported through a commercial smartwatch, customized phone app, and web-based platform.

This study exposed both the strengths and weaknesses of InteractiveClinics. Most children and their parents liked the watch and web-based platform, finding it easy to learn and simple to use. They were satisfied with the pain and physical activity module, and no adverse events were reported. However, there was also the need for improvements. Children identified usability and acceptability barriers that hindered uptake, particularly in the phone app. This finding is important because user feedback can be vastly different from how the intervention was planned to be used [70]. To improve InteractiveClinics*,* children wanted a unique in-app experience through more personalization, simplification, flexibility, conferment, comment fields, graphical illustrations, a breakdown of metrics, and interconnections.

### Suggestive Improvements

The importance of personalization is that it provides children with choice, autonomy, and ownership when using technology [71]. This is achieved by enabling children to customize the app features to suit their needs [72] and providing more feedback, on an individual level [73].

Parents also requested more simplification by removing all the buttons that were not relevant to their child. Other studies have also requested removing any complexities. DeForte et al [74], for example, reported children only wanted simple buttons. This would also support a child with a disability or low level of literacy, improving their ability to navigate the app [75].

In addition, the app needed to be more flexible. Children wanted to be able to change their medication response times within the app, earlier or later than the previously selected notification times given to the research team (SB). Rather than being reliant on the research team to update their medications, they wanted to adjust the response times to work around their activities. Interestingly, a systematic review of medication apps for adults, reported that the main components that foster medication adherence were reminders and importantly, medication-tracking histories [76]. Therefore, future changes will be made to InteractiveClinics to provide users control over their notification reminders, to ensure an accurate medication history is recorded.

Further, children wanted more from the pain module; instead of the numerical scale confirming their pain recording, they wanted more exciting conferment. In contrast, children with cancer pain in a phone app study requested the numerical scale instead of the pediatric face scale using smiley faces [77]. Therefore, it was not surprising then when children suggested more engaging gamification. Yet, very few studies have considered gamification mechanics as part of their pain assessment, despite the evidence suggesting they are liked and can increase motivation [78]. Indeed, this is certainly an area in need of further research.

There was also an overwhelming response from parents to include comment fields to record additional information such as medication-induced side effects. This is important because a recent study identified that two-thirds of children with JIA, within 1 year of diagnosis, experience side effects that have an impact on their life [79]. Understandably, concerns about these side effects can result in the termination of their treatment, posing the risk of increasing disease activity [80]. Therefore, it is imperative for InteractiveClinics to collect detailed input from children on what occurs between pediatric rheumatology appointments, to gain a more comprehensive overview of disease activity and adverse events [81], enabling timely re-evaluation and mitigation strategies to be put into place, rather than merely relying on the assessment of pain and active joint count at each visit [82]. Permitting suitable changes to treatment such as prescribing folate to reduce methotrexate-induced nausea [17,83,84] or brain fog [85], changing the route of administration [17,84], splitting doses, or altering the rate of absorbency [20] can reduce the risk of disease burden and further polypharmacy in adulthood [19].

For the physical activity module, children reported wanting more graphical illustrations representing their physical activity. According to the World Health Organization, children between the ages of 10 to 18 years are in a phase of life between childhood and adulthood where health behavior can be greatly influenced. Therefore, they are in need of age-appropriate comprehensive information [86], as they are active agents in their own development [87]. A similar study that used an activity tracker, app, and web-based profile also reported the need for more detailed feedback. Children, instead of a similar traffic light system, wanted the see the actual step count [88] (in other words, the breakdown of metrics).

To improve the app and web-based platforms’ usefulness, children also wanted to see interconnections between the medication adherence module, pain level module, and physical activity module to, for example, determine the impact pain or medications may have on physical activity levels. This is important because each of these modifiable risk factors can affect another [15,49]. Demonstrating a correlation between this information could also be useful for the pediatric rheumatology team to better understand disease progression or remission, therefore facilitating adjustments to treatment accordingly, and also to avoid the impact JIA can have on development, physical function, and health-related quality of life [15,49].

### Clinical Importance

An important clinical finding in this study was parental endorsement. Parents, in contrast to children, felt InteractiveClinics was useful in addressing their child’s problems by helping them understand the effects JIA had on their children. This is an important finding because parents’ narratives of their child’s condition often remain unmet [89]. By gaining self-awareness, the parent may gain a more insightful understanding of their child’s status on treatment that may be interfering with their progress [89]. In addition, parents then examine their own views and behaviors that can also contribute either positively or negatively to their child’s health outcomes, because children are observational learners [90]. For example, a parent’s fear and catastrophizing can result in protective behaviors and avoidance of treatments, therefore impairing their child’s functional ability [90], suggesting the importance of digital health care in educating and empowering parents.

Overall, the children and parents included in this study, through their own lived experience, were incredible collaborators in improving the usability of InteractiveClinics, greatly extending our understanding of the unique needs of children with JIA. This is important, as there is criticism toward the current “treat to target” approach used in JIA management. Children and their parents are often not included when formulating treatment plans [82]. Yet, their goals of treatment are often different from those of the Pediatric Rheumatology teams because they are focused more on the present, rather than the long impact of the disease [82], emphasizing the importance digital health care can have in supporting child-centered and family-centered care [91].

### Limitations

There are several limitations that need to be considered when interpreting this study’s findings. First, this study only recruited a small convenience sample of 12 children and 12 parents, limiting generalizability. Although a small sample size (>10 participants) is typically used for usability testing[58], the suggestive inclusion to improve InteractiveClinics may not be representative of all children with JIA. Therefore, to overcome sampling bias, a larger, more diverse participant sample is needed across different demographic and geographical locations in the next stage of testing.

Also, due to the nature of this usability and acceptability study, consenting participants were actively prompted to be critical and provide written feedback on how the proposed digital health intervention could be improved. This may have limited the provision of any positive attributes.

In addition, the written feedback they provided may have not been as detailed as expected for thematic analysis. Patterns did emerge, and data saturation was reached; however, there was still a risk of research bias [69], so all authors internally reviewed and rereviewed emerging codes and themes against the original text during all stages of analysis.

### Further Research

Further research is now needed to examine the potential challenges and limitations of incorporating InteractiveClinics into clinical practice. First, this research needs to focus on device access and digital literacy, because this study supplied all the equipment needed to participate and provided ongoing technical support. Second, the level of engagement and length of adherence among participants using the intervention needs to be considered, to clearly understand whether digital health care is an effective and sustainable intervention to support chronic disease management for children and their families.

A comparative analysis is also needed to compare InteractiveClinics to other digital health interventions targeting pediatric chronic disease. This would help with positioning the intervention within the broader digital health landscape and identifying any unique benefits that may be offered over existing tools, in order to find supportive and effective digital health solutions.

Interestingly, for JIA, 2 recent systematic reviews identified no similar digital health interventions that have used a multimodal approach to support chronic disease management [43,92]. In fact, most interventions were still at an early stage of development [43], and heterogeneity exists, making it difficult to compare their effectiveness [92]. Instead, the findings of these reviews helped to identify 3 specific areas that are needed in JIA management—symptom monitoring, physical activity promotion, and self-management development [92]—which were used to support the development of the 3 modules included in InteractiveClinics: pain level, physical activity level, and medication adherence. It is also important to note that no studies directly targeted medication adherence [43,92], yet early aggressive pharmacological treatment and the monitoring of side effects are keystones in JIA treatment [91].

Now, the future direction of InteractiveClinics is to use the feedback gained from the children and parents within this study to improve the usability of the intervention. The World Health Organization clearly reinforces the importance of doing this before commencing costly trials [58]. Then, the next step is to test the intervention’s efficacy and effectiveness [58]. This will be achieved through conducting a pilot randomized controlled trial, which will remove the sampling bias identified in this study and determine the intervention’s effectiveness on health outcomes for children with JIA and children living with other chronic conditions.

### Conclusion

Most children and their parents liked using the smartwatch and web-based platform; they found it easy to learn and simple to use. They were also satisfied with the pain and physical activity modules. However, usability and acceptability barriers that hindered uptake were identified in the phone app and medication module. Children sought a unique in-app experience, and their suggestive improvements included more personalization within the app; simplification by removing all nonrelevant links; flexibility in response times; improved conferment through gamification; additional comment fields for the input of more data, such as medication side effects or pain-related symptoms; more detailed graphical illustrations of the physical activity module, including a breakdown of metrics; and importantly, interconnections between the modules, because medication adherence, pain levels, and physical activity can each influence the other. Further research is now needed to ensure these inclusions are combined with standardized comprehensive assessments and evidence-based behavior change strategies to promote user adoption and advancement of new, validated digital health interventions in pediatric rheumatology clinical care.

## Abbreviations

eVas: electronic visual analog scale
JIA: juvenile idiopathic arthritis
mHealth: mobile health
